# Role of metabolomics in the delivery of precision nutrition

**DOI:** 10.1016/j.redox.2023.102808

**Published:** 2023-07-05

**Authors:** Lorraine Brennan, Baukje de Roos

**Affiliations:** a Institute of Food and Health and Conway Institute, UCD School of Agriculture and Food Science, UCD, Belfield, Dublin 4, Ireland; b The Rowett Institute, University of Aberdeen, Foresterhill, Aberdeen, AB25 2ZD, United Kingdom

**Keywords:** Metabolomics, Metabolic profiling, Precision nutrition, Personalised nutrition, Biomarkers

## Abstract

Precision nutrition aims to deliver personalised dietary advice to individuals based on their personal genetics, metabolism and dietary/environmental exposures. Recent advances have demonstrated promise for the use of omic technologies for furthering the field of precision nutrition. Metabolomics in particular is highly attractive as measurement of metabolites can capture information on food intake, levels of bioactive compounds and the impact of diets on endogenous metabolism. These aspects contain useful information for precision nutrition. Furthermore using metabolomic profiles to identify subgroups or metabotypes is attractive for the delivery of personalised dietary advice. Combining metabolomic derived metabolites with other parameters in prediction models is also an exciting avenue for understanding and predicting response to dietary interventions. Examples include but not limited to role of one carbon metabolism and associated co-factors in blood pressure response. Overall, while evidence exists for potential in this field there are also many unanswered questions. Addressing these and clearly demonstrating that precision nutrition approaches enable adherence to healthier diets and improvements in health will be key in the near future.

## 1 Introduction

The recent data from the Global Burden of Disease Study highlighted the important role poor diet plays on non-communicable disease (NCD) mortality and morbidity [1]. Importantly, the data demonstrated that suboptimal diet is responsible for more deaths than other risk factors. Additionally, high rates of obesity and diet related diseases in children are reported [2]. Consequently, there is an urgent need to develop innovative ways to improve diets and enable shifts to higher quality diets. This need coupled with the increasing evidence that individuals respond differently to diets has highlighted the requirement to move away from “one size fits all” approaches [[3], [4], [5]]. With the emergence of omics technologies the concept of precision nutrition emerged where combining different biological data can help understand variability in response to diets [[5], [6], [7]]. While both “personalised nutrition” and “precision nutrition” terms are used interchangeably by some and distinct differences defined by others, in the present review we use precision nutrition in the broadest terms.

The omic technologies have contributed to the development of precision nutrition and offer great potential as the field moves forward not only to understand variability in response to diet but also to predict such responses. Metabolomics is the study of small molecules called metabolites and application of metabolomics in the context of precision nutrition is the focus of this review [8]. Metabolomics is suited to exploit the study of food by measuring both exogenous and endogenous metabolites [9]. Focusing on exogenous metabolites can capture information on food intake while measuring the endogenous metabolome can inform how diet impacts on metabolic pathways [9,10]. This dual aspect of metabolomics can play a pivotal role in the development and delivery of precision nutrition. In this review, we focus on key concepts and studies that employ metabolomics/biomarker measurements to progress the precision nutrition field. In particular, we focus on areas where metabolomics has played a key role such as food intake biomarkers, metabolic phenoptyping and response to interventions.

## 2 Biomarkers of food intake – role in precision nutrition

The limitations associated with current methods for assessment of food intake are well documented in the literature [[11], [12], [13], [14]]. Metabolomics derived biomarkers of food intake have potential to address some of these limitations. These metabolites reflect food intake and in some instances can also reflect the quantity of food consumed. A recent review of the literature examined data in relation to 67 foods and food components and reported 347 potential biomarkers [15]. From this review, biomarkers for wholegrains, soy and sugar were the most reliable. However, a full validation of all potential biomarkers is needed to ensure that we are moving towards improved dietary assessment. Such a validation scheme was developed by the European Consortium, FoodBall [16]. The validation criteria included assessment of biological plausibility, time–response, dose–response, robustness, reliability, stability, and analytical performance of the method used to measure them. Furthermore, a series of systematic reviews were conducted to examine biomarkers of a range of different foods [17,18]. While many putative biomarkers were identified for foods including citrus, red meat, coffee, green leafy vegetables, cereal foods, apple, pear and stone fruit, there are relatively few that are fully validated [[17], [18], [19], [20], [21], [22]].

For biomarkers to be useful in assessment of dietary intake for delivery of precision nutrition it is imperative that large scale efforts are made to validate food intake biomarkers both at an individual and population level [10,23]. Working through the validation criteria for Food intake biomarkers will remove biomarkers that are not suitable and may be influenced by factors other than food intake. Understanding the impact of factors such as age, BMI and ethnicity on biomarker levels is important to drive forward their potential use [24]. Furthermore, we need to progress the use of food intake biomarkers so that they have added value over traditional self-reported approaches or can be used in conjunction with such approaches. Examples of where metabolomic derived biomarkers were used to calibrate self-reported data exists. In a recent study a combination of urinary and serum biomarkers for red meat were used to develop calibration equations in a biomarker study with 450 participants. These calibration equations were then used to adjust the self-reported data from FFQs in a larger study to examine associated of intake with cardiovascular disease, cancer, and diabetes incidence [25]. While a high-meat dietary pattern was associated with higher chronic disease risks, the associations were attributable to high-fat, high-energy and high-sodium that occur in a high red-meat diet rather than to the meat in this population group. The employment of the biomarkers enables a fresh look at the associations between dietary factors and disease risks and outcomes. Work in our research group developed calibration curves using a controlled dietary intervention to allow determination of citrus intake from urinary proline betaine [26]. We further developed regression calibration curves using dietary data from 4-day food records and biomarker data which could be used to correct self-reported data [27]. Both these examples demonstrate the potential of food intake biomarkers to improve the accuracy of dietary assessment which in turn can be used to assess individuals’ diet prior to the delivery of personalised advice. Accurately, assessing current intake could aid in the prioritisation of foods to focus on for personalised advice.

Concomitant with the development of the concepts in relation to specific food intake biomarkers is the use of panels of biomarkers to assess dietary patterns. There are several studies that have used biomarkers to examine adherence to established dietary patterns such as the Mediterranean diet [[28], [29], [30]]. A series of serum metabolite levels in postmenopausal women were used to distinguish between low and high adherence to four healthy diet scores [31]. Other studies have similarly found associations between metabolites and predefined dietary patterns [30,[32], [33], [34]]. More recently, urinary profiles were linked with the Alternative Healthy Eating Index [35]. While measurement of adherence to dietary patterns is interesting, the assessment of the links with disease risk represents exciting possibilities for the development of precision nutrition. A metabolites signature including 67 metabolites was associated with adherence to the Mediterranean diet in a Spanish population and replicated in US cohorts. Then in prospective analysis, the metabolite signature was predictive of CVD risk [36]. In a recent study a total of eight metabolites (mannose, γ/β-tocopherol, N1-methylinosine, pyrraline and four amino acids) were inversely associated with three healthy dietary patterns [37]. These metabolites were associated with worse cardiometabolic traits and elevated diabetes risk, indicating that targeting a reduction in these metabolites could be a successful precision nutrition strategy.

While adherence to pre-defined dietary patterns can inform us about the dietary quality of an individual, there is also interest in using panels of biomarkers to classify individuals into dietary patterns. The advantage of such an approach is that is removes the reliance on self-reported data and therefore reduces the burden on participants. Classification into a dietary pattern could be followed by personalised dietary advice. Evidence exists to support the potential of combination of biomarkers to classify individuals into dietary patterns. Using urinary metabolomics data individuals were classified into dietary patterns and the classification was validated in independent studies [38]. Work in our research group also employed the urinary NMR profile to classify individuals into four dietary patterns and replication was achieved in a separate population group which also demonstrated good reproducibility over four timepoints.

**Fig. 1:**
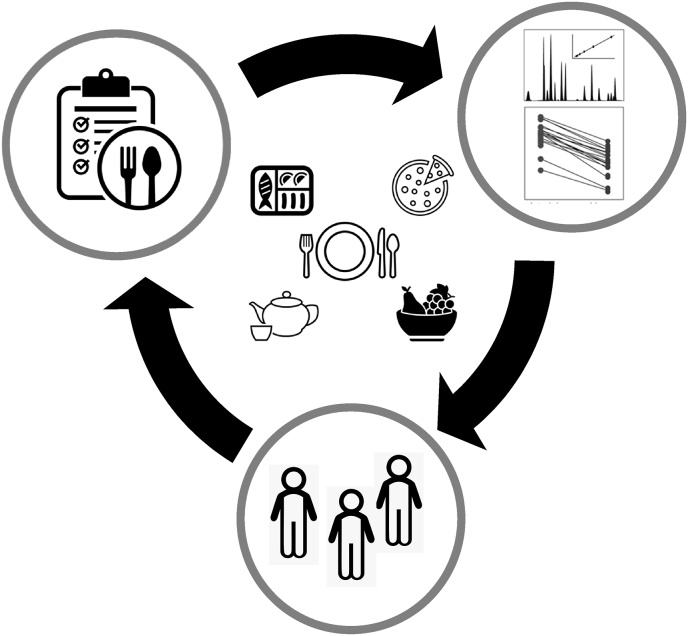
**Role of metabolomics in the assessment of dietary intake.** Metabolomics based biomarkers can give objective information on food intake, and therefore improve the accuracy of dietary assessment acquired through dietary questionnaires,. Furthermore, metabolomics can measure an individuals' exposure to bioactive compounds. Therefore, metabolomics approaches can aid the development and implementation of precision nutrition approaches.

There is interest in using metabolomic biomarkers for assessment of exposure to bioactive compounds such as polyphenols. Dietary polyphenols are typically referred to as anti-oxidants, as *in vitro* these compounds can scavenge reactive oxygen, nitrogen, and chlorine species, whilst chelating metal ions that could promote oxidation reactions. However, the anti-oxidant properties of polyphenols are perhaps less relevant *in vivo* due to their low concentrations in plasma, and their rapid metabolism by liver enzymes and gut bacteria. Also, their metabolites may not have the same level of antioxidant activity. Instead, dietary polyphenols may act as pro-oxidants by activating the transcription factors of nuclear factor erythroid 2 related factor 2 (Nrf-2) and heat shock factor (HSF), as shown in several *vitro* and *in vivo* studies, and reviewed by us [39]. This supports a role for dietary polyphenols in enhancing the production of anti-oxidant enzymes and heat shock proteins to protect against the potential damage by ROS. Measurement of polyphenols and the derived metabolites offers the potential to assess exposure and can help address and understand the interindividual variation in response to polyphenol interventions [40]. Furthermore, consumption of bioactive compounds like polyphenols are believed to affect disease outcomes depending on their concentration in the diet, on genetic factors that determine enzyme activity, on gut microbiota composition and on lifestyle, which are all highly individualized factors and therefore important in the context of precision nutrition [41]. Collectively, the above examples illustrate the power of metabolomics in terms of assessment of food intake which in turn has potential for development and implementation of precision nutrition (Fig. 1).

## 3 Metabotyping for precision nutrition

**Fig. 2:**
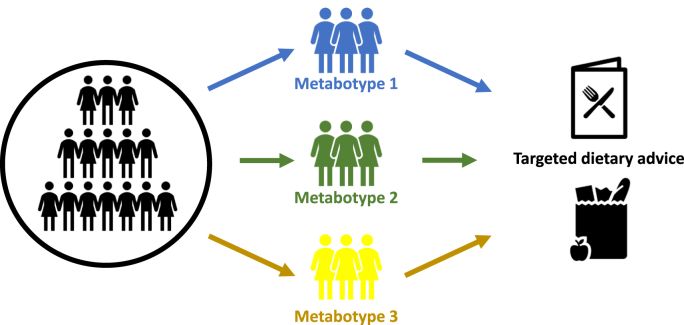
**Role of metabotyping in the development of targeted dietary advise.** Metabotypes are subgroups of metabolically similar individuals. The use of metabolomics to define subgroups, and the development and delivery of tailored dietary advice to these subgroups, is a tool for delivery of precision nutrition.

In recent years the concept of metabotypes has emerged which involves using metabolic parameters to group individuals into subgroups with similar metabolic profiles. Metabolomics plays a key role in either defining these subgroups/metabotypes or supporting the definition of the metabotypes. Interest in metabotypes emerged initially through research demonstrating that metabotypes had differential response to diets and interventions [[42], [43], [44]]. Developing precision nutrition approaches based on metabotypes is an attractive approach to deliver advice that is tailored to the metabolic profile of the individuals (Fig. 2).

A recently published study examined the ability of metabotypes to deliver nutrition advice. During a 12 week randomised controlled intervention the PERSON study delivered advice according to metabotypes of tissue specific insulin resistance [45]. Using an oral glucose tolerance test (OGTT), individuals were classified as muscle insulin resistant or liver insulin resistant. For each metabotypes participants were randomised to a diet that was optimal or suboptimal for their phenotype with the hypothesis that those randomised to the optimal diet would have improvements in the primary (the disposition index) and secondary (insulin sensitivity, glucose homeostasis, serum triacylglycerol, and C-reactive protein) outcomes. While no changes in the primary outcome were observed, significant improvements in the secondary outcomes were reported. Furthermore, the improvements were for individuals who received the suboptimal diet highlighting the complexity of delivering precision nutrition. Nonetheless, the study clearly supports the concept that modulation of diet based on metabotypes can produce more pronounced clinically relevant improvements in cardiometabolic health.

Delivery of tailored dietary advice to a general population group through use of metabotypes is an attractive approach. Using four routinely measured markers of metabolic health (triacyglycerol, cholesterol, HDL-cholesterol and glucose), a total of three metabotypes were identified in a population [46,47]. Subsequently, a framework was developed to deliver nutrition advice to each metabotype [47]. The metabotypes were successfully replicated in the German cohort KORA and the incidence of cardiometabolic diseases differed across the metabotypes supporting the use of the framework to delivery tailored advice [43]. The ability of this framework to deliver personalised nutrition is currently under investigation using a 12-week randomised controlled trial (RCT) (n = 107) [48]. The primary outcome will determine if the metabotype approach is an effective mechanism of improving diet quality and metabolic health parameters.

The PREVENTOMICS platform was developed to deliver personalised advice to overweight and obese individuals [49,50]. Employing 52 urine and blood biomarkers in conjunction with 35 SNPs, the algorithm calculated scores for each individual based on five metabolic processes including oxidative stress, inflammation, carbohydrate metabolism, lipid metabolism and gut microbiota metabolism. In a 10-week RCT, the platform was tested for its ability to improve fat mass compared to generic dietary advice. However, following the intervention, both the personalised and generic groups improved fat mass, body weight, diastolic blood pressure and metabolic health biomarkers. While following the metabotype diet did not improve the outcomes further, work is needed to better define/refine the metabotypes. It is also possible that the study duration was not long enough to see the added benefit of advice via the personalised approach.

Collectively these studies highlight the potential of metabotypes for delivery of precision nutrition. Further work is needed to demonstrate the efficacy of the approach and to understand the mechanistic underpinnings. Rather than focus on a specific set of metabolites relevant for a particular metabotype the important aspect of the work that has emerged to date is the overall framework for using metabotyping. Using the approach to sub group the population group and then tailor the advice according to the sub-group is the key underpinning concept. Future work is now needed to develop these concepts further and demonstrate the efficacy of tailoring dietary advice based on sub-groups/metabotypes within a population.

## 4 Use of metabolomics to predict response to dietary intervention

**Fig. 3:**
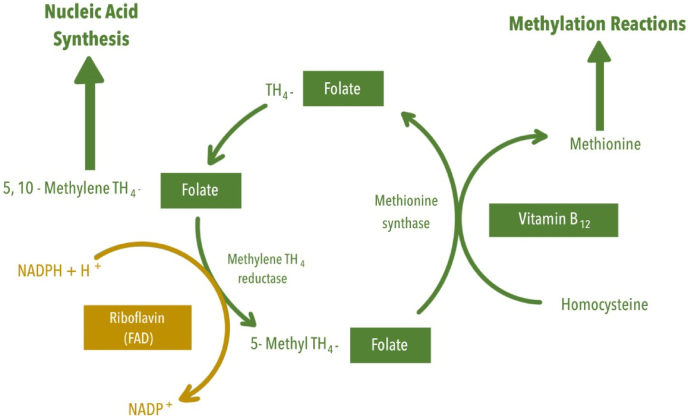
**Riboflavin and one carbon metabolism**. The B vitamin riboflavin, in its co-enzymatic form FAD, is required as a cofactor for methylenetetrahydrofolate reductase (MTHFR), which catalyses the reduction of 5,10 methylene THF to 5-methyl THF. 5-methyl THF is required by methionine synthase for the vitamin B12–dependent conversion of homocysteine to methionine, which, once activated by ATP, forms the methyl donor S-adenosylmethionine. Decreased MTHFR enzyme activity is evident in individuals where high blood pressure is associated with homozygosity for the common C677T polymorphism in the *MTHFR* gene, but supplementation with riboflavin appears to stabilise MTHFR enzyme activity.

The field of precision nutrition aims to determine the factors associated with differences in response to dietary interventions. There are a small number of elegant studies which have, following careful consideration of mechanistical pathways, established that response to a diet, or weight loss intervention, could be predicted by one or two single factors. For example, microbial enterotypes are characterized by distinct digestive functions with preference for specific dietary substrates, resulting in short-chain fatty acids that may influence energy balance in the host. Consequently, the enterotype may have the ability to affect an individuals’ ability to lose weight. This was demonstrated in a study where stratification of individuals according to two microbial enterotypes, e.g. dominance of either Prevotella or Bacteroides, helped to predict weight loss responses following an average Danish diet or a high-fibre new Nordic diet. The high-fibre diet seemed to optimize weight loss among Prevotella-enterotype subjects but not among Bacteroides-enterotype subjects [51]. Another example relates to predicting the blood pressure lowering response in those where high blood pressure is associated with homozygosity for the common C677T polymorphism in the *MTHFR* gene. The homozygous TT variant is associated with a decreased enzyme activity of methylenetetrahydrofolate reductase (MTHFR). This is due to the loss of the B-vitamin riboflavin, which, as the precursor of the co-enzyme and electron carrier flavin adenine dinucleotide (FAD), is required as a cofactor for MTHFR (Fig. 3). Riboflavin appears to stabilise MTHFR *in vivo*, and a range of randomized controlled studies have subsequently shown that riboflavin supplementation significantly reduces systolic blood pressure by 5-13 mmHg, specifically in individuals with the MTHFR 677 TT genotype [52]. A third study, already discussed above, validated previous observations that the presence of specific insulin-resistant phenotypes may predict cardiometabolic responsiveness to specific diets differing in dietary macronutrient composition, e.g. a high MUFA diet or a low-fat high complex carbohydrate diet [45].

However, prediction of response is usually more complex. To enable the identification of predictive factors, we typically need large studies, with hundreds of participants, that examine a whole range of biomarkers which may help to explain the differences in response between participants, including genetics, the participant's gut microbiome, body composition, hyperlipidaemic and diabetic phenotypes, and a range of metabolites indicative of, for example, glucose or protein metabolism, as identified through metabolomics. More recently, studies have started to use more advanced statistical approaches and machine-learning algorithms to exploit the inter-individual differences in response to foods, meals and diets, for example in relation to postprandial (post-meal) glycaemic and lipid responses, based on a much wider range of individual genetic and/or phenotypic traits, often obtained by the application of omics technologies. Two landmark studies have pioneered the field of precision nutrition and predicting individual responses dietary intake. The first study, performed in an Israelian population by Zeevi and colleagues [6], measured continuous glucose responses to 46,898 meals in 800 participants. Typically, studies report a high variability in the glucose response to identical meals between individuals [7,53]. But by integrating individual data on blood clinical chemistry outcomes, dietary habits, anthropometrics, physical activity and gut microbiota, machine-learning algorithms could accurately predict individual postprandial glycaemic responses to a standardised breakfast. Individual BMI, HbA1c, fasting glucose, and age were, as expected, strongly correlated with the postprandial response to a standardized breakfasts and real-time meals, but the study also found significant positive correlations between the individual postprandial response of participants and some of their clinical data, such as HbA1c, alanine aminotransferase, CRP, and their gut microbiome. These results suggest that postprandial glycaemic responses are associated with multiple and diverse factors, including factors related to meal content (carbohydrate, fat, fibre, alcohol, and sodium), or unrelated to meal content (time that passed since last sleeping, cholesterol levels, HbA1c%, and microbiome) [6]. The model of personalised algorithms developed for this particular study was subsequently applied in a second study to design a personalised diet for Israelian adults with newly diagnosed Type2 Diabetes Mellitus, aiming to improve personal postprandial glucose responses and metabolic health, as compared with the commonly recommended Mediterranean-style diet. The authors found that the personalised diet significant lowered levels of continuous glucose measurement-based measures, including the average postprandial glucose response and mean glucose levels. The personalised diet also significantly improved multiple metabolic health parameters, including HbA1c, fasting glucose, and triglycerides, and in over half of the participants, diabetes remission was observed [54]. The model of personalised algorithms, as developed by Zeevi et al. [6], was also applied in the Personal Diet Study in 1999 overweight and obese American adults with abnormal glucose metabolism and obesity. Participants were either consuming a low-fat diet, or a personalised diet where meal choices were coded green, yellow or red based on individual estimated postprandial glucose responses. In this latter group, participants were instructed to make different food choices or substitutions to change a yellow or a red score to a green score. Participants in both groups lost weight, although not to the extent that it could be considered clinically meaningful. The personalised diet targeting a reduction in PPGR did not result in greater weight loss compared with a low-fat diet, after 6 months [55]. The second study, performed in a UK and USA population [5], assessed a range of postprandial metabolic responses in 1002 twins and unrelated healthy adults. They observed a large interindividual variability in postprandial responses of blood triglycerides, glucose and insulin following identical and standardised muffin meals. Interestingly, this study found that different person-specific factors had a higher or lower degree of influence on each of these postprandial responses. For example, meal composition, genetics, meal context, serum glycaemic markers and the gut microbiome had the greatest influence on postprandial glucose levels, whereas serum lipids and fasting triglycerides were the main determinants of postprandial triglycerides, in both cases explaining nearly 50% of variance. Overall, genetics had relatively little (<10%) impact on responsiveness to meal composition and in this study, the role of metabolomic phenotyping appeared more useful in the delivery of precision nutrition [5]. Overall, the potential role of heritability in predicting outcomes appears to vary depending on the outcomes being considered; twin studies have shown that heritability estimates for adherence to dietary indices varied between 10 and 43% [56], whereas environmental factors such as diet and anthropometric measures are much more important in shaping human gut microbiota than host genetics [57]. On the other hand, for Type 2 Diabetes prevalence, heritability is believed to be as high as 72%, and the impact of environmental factors is much lower as evidenced by the low number of discordant monozygotic pairs for Type 2 Diabetes in the DISCOTWIN study [58].

Previous approaches, which attempted to link postprandial glycaemic responses to the intrinsic properties of a consumed food, such as is done with using the glycaemic index model [59], are now superseded by evidence from, for example, the studies above that, whilst individual postprandial glucose and lipid responses are still strongly dependent on factors like meal composition, endogenous phenotypic characteristics, such as serum glycaemic markers and the gut microbiome, also play an important role when predicting individual response [5,6,55]. This means that it is possible to develop personalised diet strategies to predict and therefore better manage postprandial glucose and triglyceride responses, at least in populations that were represented in these two large studies. It would be interesting to see, however, whether use of such algorithms provide comparable predictive ability in other populations.

Personalised strategies to better exploit the potential of diet or dietary components to improve health outcomes depend on our ability to identify ‘response’ to diet in intervention studies, and based on that, identify which characteristics determine whether one person is a ‘responder’ and another person is not. Only recently have studies started to look into identifying responders and non-responders to intervention in a more comprehensive and considered way, in an attempt to push forward the field of personalised and precision nutrition, as highlighted below. Many of these studies have benefitted from the richness of omics datasets, especially when involving metabolomics, to better define the individual phenotype. For example, a recent study characterised glycaemic responders and non-responders to a low-caloric diet, based on significant improvements in visceral fat, overall and tissue-specific insulin resistance following a low caloric diet (800 kcal/d) for 8 weeks. The analysis was performed in 375 participants of the DiOGenes multicentre randomised controlled dietary intervention study who had lost >8% body weight. All had similar body composition, glycaemic control, adipose tissue transcriptomics and levels of plasma ketone bodies at baseline. However, integrative analyses of plasma Somalogic proteomics, LC-MS lipidomics, NMR metabolomics and clinical biochemistry analysis identified a plasma omics model of baseline parameters that could predict non-responders for weight loss and insulin sensitivity improvement better than clinical models. The study elegantly demonstrated that differences in responsiveness may be due to de novo lipogenesis, keto-metabolism and lipoprotein metabolism, suggesting an important role for adipose and liver tissue in metabolic improvement following low-calorie intervention [60].

Another study characterised individual responses in postprandial glucose levels, and the inter- and intra-individual reproducibility of postprandial glucose responses, in overweight and obese adults upon consumption of hydrolysed milk proteins drinks. Independent t-tests were used to explore if individuals were responders or non-responders to either one of two casein hydrolysates, compared with consumption of an intact caseinate supplement, at an individual participant level at P < 0.05. The ingestion of one specific casein hydrolysate successfully reduced the postprandial glucose response at the group level. However, at an individual level, only 3 participants were classified as ‘responders’. This could be linked to inter-individual coefficients of variation being significantly higher than the intra-individual coefficients of variation, in responses to dietary intervention [7].

Two further studies recently assessed inter-individual variability in response to protein and fish oil supplementation in older adults at risk of sarcopenia [61], and how inter-individual variability in response to fish oil supplements could be used to predict the triglyceride-lowering response in healthy adults [62]. The first of these two studies set out to test the interindividual variability in responses in appendicular lean mass, leg strength, timed up-and-go, and serum triglyceride concentrations, to supplementation with leucine-enriched protein, leucine-enriched protein plus fish oil, or a control supplement, in older adults at risk for sarcopenia. In order to determine ‘responsiveness’, inter-individual variability in response to supplementation was estimated by comparing the standard deviation of individual responses with the minimally clinically important difference. Responsiveness to supplementation was then assessed as clinically meaningful interindividual variability, e.g. when the standard deviation of the individual response exceeded the minimally clinically important difference in the main outcomes. This methodological approach indicated that there was minimal inter-individual variability in changes in appendicular lean mass, leg strength, timed up-and-go, and serum triglyceride concentrations in response to protein and fish oil supplementation in older adults at risk of sarcopenia [61]. For the second study, we used data from the placebo-controlled crossover FINGEN study, assessing the effects of fish oil supplements in 312 healthy individuals [63], to predict change in concentrations of plasma triglycerides and in plasma levels of the main fish oil fatty acids eicosapentaenoic acid (EPA) + docosahexaenoic acid (DHA). We developed variable selection models, based on forward and backward stepwise selection, LASSO and the Boruta algorithm, to show that fish oil supplementation led to a greater lowering in triglycerides in those with lower pre-intervention levels of plasma insulin, LDL cholesterol, and saturated fat consumption, and higher pre-intervention levels of plasma triglycerides, and serum IL-10 and VCAM-1 levels. The models also found greater increases in plasma levels of EPA + DHA in those who were older and were female. This study highlights the opportunities for secondary analysis of large trial datasets to identify those who are more likely to benefit from intervention, in this case fish oil supplementation, in terms of relevant physiological outcomes [62].

There has been discussion on how best to identify response in intervention studies. Some studies have dichotomise continuous physiological outcomes into “responders” or “non-responders”, with responders being participants in a study group whose individual response is above or below a certain response threshold deemed to be clinically important. However, it may not be appropriate to label someone as a responder or non-responder from a single pair of values (e.g. a baseline versus end value, representing a change from baseline); as responses may vary from occasion to occasion [64]. Indeed, pre-to-post within subject variability, due to within-person variability because of fluctuations in physiology, and due to technological variability when measuring physiological markers [65], can introduce significant variation between consecutive measurements. Another issue with dichotomisation is that responses could potentially be explained by ‘regression to the mean’, especially when baseline values in, for example blood pressure or plasma lipids, were particularly high [66]. Using outcomes on a continuous scale rather than being dichotomised into responders and non-responders to intervention, as done by Potter et al. [62], would maximise statistical power [64,66]. Furthermore, it has been proposed that a less biased and more informative approach should use the standard deviation of individual responses to estimate the chance a new person from the population of interest will be a responder [66].

N-of-1 trials would make predictions for individuals more accurate [64]. Such studies repeatedly assess the response to one or multiple treatments in the same person. Repeated measurements are increasingly being facilitated by a growing number of outcomes that can be measured automatically, outside of the clinic, via electronic devices, such as continuous glucose measurements. N-of-1 designs can be applied to both short-term physiological and clinical studies, as well as longer-term studies to assess how everyday behaviours affect individual health. This may help to reveal novel associations between participant characteristics and health outcomes, with repeated measures providing power and precision to accurately determine an individual's health status [62].

A recent series of N-of-1 studies aimed to investigate the individual variability in postprandial glycaemic response when eating diets with different proportions of dietary fat and carbohydrates, in apparently healthy Chinese adults. The authors applied a Bayesian analysis model to calculate the posterior probability of a clinically meaningful difference in maximum postprandial glucose, mean amplitude of glycaemic excursions, and the total area under the CGM curve from 00:00 to 24:00 h, between the 6-day intervention periods where participants received either the low fat-high carbohydrate or high fat-low carbohydrate diets, at the individual level. Amongst those with a posterior probability >80%, 9 of the 30 participants were identified as high-carbohydrate responders whilst 6 of the 30 participants were identified as high-fat responders. Analyses of the Bayesian-aggregated n-of-1 trials among all participants showed a relatively low posterior probability of reaching a clinically meaningful difference of the 3 outcomes between low fat-high carbohydrate and high fat-low carbohydrate diets [67].

**Fig. 4:**
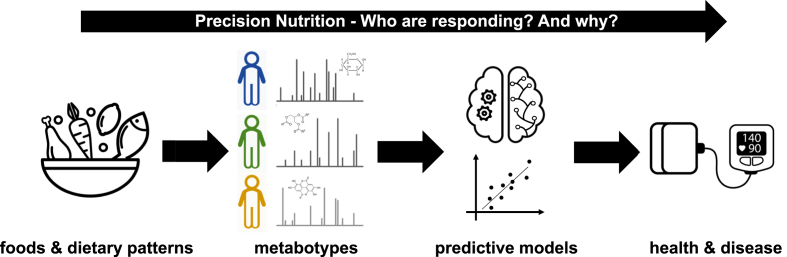
**Predicting response to dietary interventions**. The development of metabotyes, and predictive models encompassing metabolomic profiles, can be used to predict which individuals respond to specific dietary interventions in terms of important health outcomes like blood pressure and other cardiovascular risk factors. These precision nutrition approaches will enable the tailoring of dietary advice to individuals with the ultimate goal of improving individual health outcomes.

All of these studies show how implementation of omics technologies, especially those that relate to continuous glucose monitoring, and application of appropriate statistical methods to rich and larger datasets, can develop our knowledge of the factors underpinning the heterogeneity in physiological responses due to dietary interventions (Fig. 4). Such approaches can provide a useful tool for precision nutrition, and in the tailoring of dietary recommendations, for different population groups. Development of specific targeted metabolomic assays that measure specific metabolites in a quantitative fashion will enable testing of approaches across multiple studies and study populations.

## 5 Future perspectives

The area of precision nutrition, and the concepts underpinning it's research, have only recently started to attract a lot more attention. The opportunities that omics technologies offer, especially metabolomics, to broaden our understanding of dietary intake and how this links to disease development, as well as to evaluate individual responsiveness based on metabotypes, and/or other factors including someone's genetics, their gut microbiome, body composition, and metabolites indicative of glucose or protein metabolism, offer important improvements on how we can provide personalised and more precise dietary advice. Advanced statistical and AI approaches play an increasingly important role in this development, especially in relation to the integration of multi-omics datasets to define metabotypes and predict individual response, as pioneered in a few precision nutrition studies although it is recommended that such AI approaches should be applied carefully and transparently [64]. Furthermore, careful consideration is needed to merge the developments with the updating of national dietary guidelines.

However, whilst the studies performed in this area thus far are promising, as outlined above, there are important knowledge gaps we need to consider. For example, will more precise dietary advice for individuals or specific population groups lead to better adherence to healthier diets, and to better individual health outcomes? The Food4Me study [68] was one of the first proof-of-principle studies suggesting that personalised approaches could lead to improved adherence and health outcomes, at least during the 6 months of the study. However, evidence from more longer-term studies, in real-life settings, is currently lacking. We also lack evidence on the cost efficacy of precision nutrition approaches in healthcare settings. The application of precision nutrition approaches could be of particular benefit to those diagnosed with chronic diseases, but only if precision nutrition approaches would prove to be good value for money in comparison to more standard and conventional pharmaceutical approaches. Finally, but importantly, most studies thus far have been performed in educated individuals recruited from a specific geographical area, with a relatively homogenous phenotype, thus limiting the generalizability of findings. It is worth noting that N-of-1 studies are ideally suited to study the effects of behavioural and environmental factors on dietary compliance and efficacy. N-of-1 studies could also be used to investigate more complex research questions and to study underrepresented groups [69].

Finally, from a methodology development viewpoint it is imperative that metabolites are measured quantitatively and using well validated methodologies to enable actionable decisions to be made. Development of novel collection devices to enable robust collection of biological samples at home will facilitate the expansion of precision nutrition to a broader population group. Ensuring that such collection devices are compatible with getting robust metabolite measurements will be key. Concomitant with this is the need to develop wearable sensors that could capture real time monitoring of a range of metabolite levels. Considerable success has been obtained using the continuous glucose monitors: expanding the measurements to include metabolites that capture more metabolic pathways should lead to significant progress in the research field of precision and personalised nutrition. In order to create public health impact, however, such methodological developments will need to be adopted by clinical and healthcare practice as part of primary and tertiary prevention strategies, and/or in a home-setting for raising health awareness and to allow monitoring of personal health. At present, the number of outcomes being analysed by personalised nutrition platforms, and therefore the quality and tailoring of personalised advice provided to users and patients, is limited and often not cost-effective. It is important, therefore, not to over-promise the potential of precision nutrition and to allow sufficient time for the field to reach scientific maturity. It is also critical to initiate and maintain clear and open dialogues between scientists, clinicians, public health bodies and “users” in order to foster trust, and to develop strategies for the field of precision nutrition to move on from general population-based dietary guidelines to include more targeted dietary advice that better predicts dietary health outcomes on an individual level, and to better suit the individual on their personalised health journey.

## Declaration of competing interest

The authors declare the following financial interests/personal relationships which may be considered as potential competing interests: Lorraine brennan reports financial support was provided by Health Research Board.

## Data Availability

No data was used for the research described in the article.
